# Physiological and Multi-Omics Integrative Analysis Provides New Insights into Tolerance to Waterlogging Stress in Sesame (*Sesamum indicum* L.)

**DOI:** 10.3390/ijms26010351

**Published:** 2025-01-03

**Authors:** Lu Zhang, Suhua Wang, Xuele Yang, Luqiu He, Liqin Hu, Rui Tang, Jiguang Li, Zhongsong Liu

**Affiliations:** 1 Agricultural College, Hunan Agricultural University, Changsha 410128, China; zhanglu240020@outlook.com; 2 Crop Research Institute, Hunan Academy of Agricultural Sciences, Changsha 410125, China; susannawang512@163.com (S.W.); yangxuele1002@163.com (X.Y.); hlq4692102@163.com (L.H.); hnliqin1003@126.com (L.H.); hunaastr@126.com (R.T.); 3 Yuelushan Laboratory, Changsha 410128, China

**Keywords:** sesame, waterlogging stress, transcriptomics, metabolomics, miRNA, multi-omics analysis

## Abstract

Plant growth and development require water, but excessive water hinders growth. Sesame (*Sesamum indicum* L.) is an important oil crop; it is drought-tolerant but sensitive to waterlogging, and its drought tolerance has been extensively studied. However, the waterlogging tolerance of sesame still has relatively few studies. In this study, two kinds of sesame, R (waterlogging-tolerant) and S (waterlogging-intolerant), were used as materials, and they were treated with waterlogging stress for 0, 24, 72, and 120 h. Physiological analysis showed that after waterlogging, sesame plants responded to stress by increasing the contents of ascorbate peroxidase (APX), glutathione (GSH), and some other antioxidants. The results of the multi-omics analysis of sesame under waterlogging stress revealed 15,652 (R) and 12,156 (S) differentially expressed genes (DEGs), 41 (R) and 47 (S) differentially expressed miRNAs (DEMis), and 896 (R) and 1036 (S) differentially accumulated metabolites (DAMs). The combined DEMi-DEG analysis that 24 DEMis regulated 114 DEGs in response to waterlogging stress. In addition, 13 hub genes and three key pathways of plant hormone signal transduction, glutathione metabolism, and glyoxylate and dicarboxylate metabolism were identified by multi-omics analysis under waterlogging stress. The results showed that sesame regulated the content of hormones and antioxidants and promoted energy conversion in the plant through the above pathways to adapt to waterlogging stress. In summary, this study further analyzed the response mechanism of sesame to waterlogging stress and provides helpful information for the breeding of plants for waterlogging tolerance and genetic improvement.

## 1. Introduction

Plant growth and development require water. Water is a key factor for plants, but excessive water can also cause severe harm [1]. However, as a result of climate change, rainfall has become more severe and unpredictable in recent decades, and the frequent occurrence of localized torrential rains and waterlogging disasters has posed great challenges to agricultural production and seriously affected food security [2]. Excessive water results in the water content in the growing environment of plants exceeding normal required levels, and oxygen content in the soil decreases, which severely affects the plants [3]. A continuously waterlogged environment will have different effects on plant growth than effects due to single occurrences of waterlogging [4]. In the early stage of waterlogging, plants grow well because of the rich dissolved oxygen in the water [5]. However, under continuous waterlogging stress, the dissolved oxygen in the water is exhausted, and the root respiration of the plant will gradually be hindered, resulting in physiological changes such as blocked nutrient transport, reactive oxygen species (ROS) damage, toxic metabolite accumulation, and weakened photosynthetic capacity, ultimately affecting plant growth and development [6].

The effects of waterlogging stress on plants are multifaceted, and there are some differences in the effects on different plants and different genotypes of the same plant [7]. In a study of waterlogging stress on citrus plants (*Citrus*), great differences were detected among different citrus genotypes, and the symptoms of damage to citrus plants appeared from 7 to 24 d, which was related to delayed oxidative damage and the accumulation of malondialdehyde (MDA) in the early stage [8]. Research on cassava (*Manihot esculenta* Grantz) waterlogging stress has shown that under waterlogging treatment, cassava can adapt to stress by reducing the chlorophyll content in leaves and increasing antioxidant enzyme activity [9]. Hormone signaling can play a role in plant response to waterlogging stress. In maize (*Zea mays*), *ZmEREB180* can increase waterlogging resistance by promoting the formation of adventitious roots through the ethylene signal transduction pathway [10]. Studies on the response of soybean (*Glycine max*) to waterlogging stress have shown that jasmonic acid (JA) and salicylic acid (SA) play important roles in the response to stress [11]. Under waterlogging stress, the demand for energy increases significantly. The *pgm* mutant in Arabidopsis (*Arabidopsis thaliana*) is very sensitive to a low-sugar environment, which increases sensitivity to waterlogging stress, and exogenous application of sugar can alleviate some phenotypes [12]. Studies have shown that plants can improve their adaptability to hypoxic environments through the *ADH* gene in response to waterlogging stress [13].

With the development of sequencing technology, the use of omics (transcriptomics, metabolomics, small RNA sequencing, lipidomics, and proteomics) technology to analyze plant responses to stress has become increasingly in-depth, and combined multi-omics analysis has also emerged [14]. In the study of lotus (*Nelumbo nucifera*) waterlogging stress, miRNA sequencing analysis revealed that *miR159*, *miR393h*, and *miR319c-3p* are involved in stress response [15]. Studies have shown that *miR398* and *miR2119* can respond to waterlogging stress by negatively regulating the expression of *CSD1* and *ADH1* in common bean and some leguminous plants [16]. In Arabidopsis, the results of multi-omics analysis revealed that *miR775-GALT9* can respond to waterlogging stress by regulating the transduction of ethylene and abscisic acid (ABA) signals [17]. In the response of Bermuda grass (*Cynodon dactylon*) to waterlogging stress, combined transcriptomics and metabolomics analysis revealed that phenylalanine metabolism and biosynthesis play important roles in the response of Bermuda grass to waterlogging stress [18]. Similar results were obtained in barley (*Hordeum vulgare*), and the *PER* and *CCR* genes play important roles in the phenylpropanoid biosynthesis pathway [19]. In the study of the response of orchardgrass (*Dactylis glomerata*) to stress, combined transcriptomics and metabolomics analysis revealed that the biosynthesis of amino acids plays an important role in waterlogging stress [20]. Combined transcriptomics and proteomics analysis revealed that the glycolysis/gluconeogenesis pathway and lignin biosynthesis are highly important in soybean waterlogging stress [21]. In summary, the combined analysis of various omics technologies is highly useful for studying the regulation of plant responses to waterlogging stress.

Sesame (*Sesamum indicum* L.) is an oil crop that originates from Africa [22]. Sesame is rich in oil, protein, vitamins, and minerals and has high nutritional value; it is an important medicinal and food homologous crop known as the ‘omnipotent nutrition bank’ [22]. This may be due to its origin; sesame has a certain degree of drought tolerance, but it is susceptible to waterlogging [23]. Studies have shown that 48 h of waterlogging treatment will cause harm to sesame, 10 d of waterlogging treatment will affect the survival, and the interaction between the production of ROS and hormone signal transduction will affect the tolerance of sesame to waterlogging [24]. It has also been found that the phenylpropanoid biosynthesis, glutathione metabolism, and nitrogen metabolism were significantly changed after waterlogging stress at the sesame flowering period [25]. In the study of waterlogging stress of 142 sesame genotypes, it was found that antioxidant capacity and anaerobic metabolism played an important role in sesame’s response to waterlogging stress [26].

In this study, transcriptome, small RNA, and metabolomics were used for multi-omics analysis to explore the regulatory mechanism of sesame in response to waterlogging stress. The results of this study are highly important for exploring the response of sesame to water stress, which is helpful for elucidating the regulatory network of the plant response to waterlogging stress and for providing valuable information for the cultivation of new waterlogging-tolerant sesame varieties.

## 2. Results

### 2.1. Analysis of the Physiological Indicators of Sesame Under Waterlogging Stress

**Figure 1:**
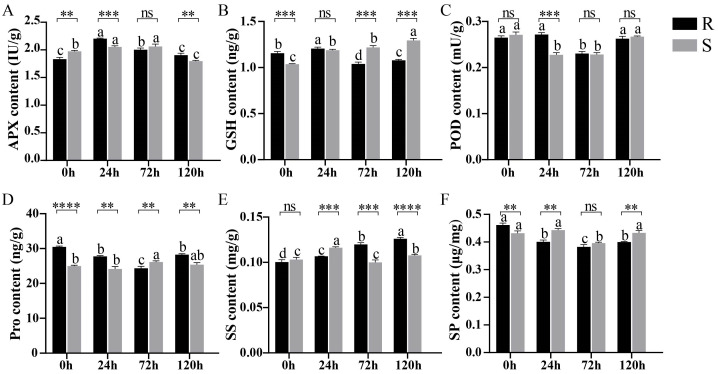
Physiological changes in different kinds of sesame under waterlogging stress. (**A**–**F**). APX, GSH, POD, Pro, SS, and SP contents in different kinds of sesame under different waterlogging stresses. APX: ascorbate peroxidase; GSH: glutathione; POD: peroxidase; Pro: proline; SS: soluble sugar; SP: soluble protein. R and S represent two types of sesame: R is waterlogging-tolerant, and S is waterlogging-intolerant. One-way ANOVA processing was used for significance analysis; different lowercase letters indicate that there are significant differences under the same sesame waterlogging stress at different times (*p* < 0.05); **, ***, and **** indicate that there is a significant difference between the two types of sesame plants at the same time (**: *p* < 0.01; ***: *p* < 0.001; ****: *p* < 0.0001); and ns indicates no significant change. Bar means the average ± SD, *n* = 3.

To understand the physiological characteristics of sesame plants under waterlogging stress, we measured a series of physiological indicators, including the contents of peroxidase (POD), ascorbate peroxidase (APX), glutathione (GSH), soluble sugar (SS), soluble protein (SP), proline (Pro), and MDA, among which the contents of POD, APX, MDA, and Pro were the key physiological indicators of waterlogging stress (Figure 1 and Figure S1). Under waterlogging stress, the APX content was first upregulated and then downregulated, and the APX content in R was significantly greater than that in S (Figure 1A). The POD content was significantly downregulated in R at 72 h of stress; this downregulation occurred earlier in S than in R, at 24 h, but the POD content remained relatively consistent in both R and S thereafter (Figure 1C). The Pro content differed between R and S under waterlogging stress, and that in R was significantly greater than that in S. The Pro content in R was significantly downregulated, whereas in S, it was slowly upregulated (Figure 1D). The MDA content increased significantly after stress, and there was no significant difference between R and S (Figure S1).

### 2.2. Analysis of Differentially Expressed Genes (DEGs) in Sesame Under Waterlogging Stress

**Figure 2:**
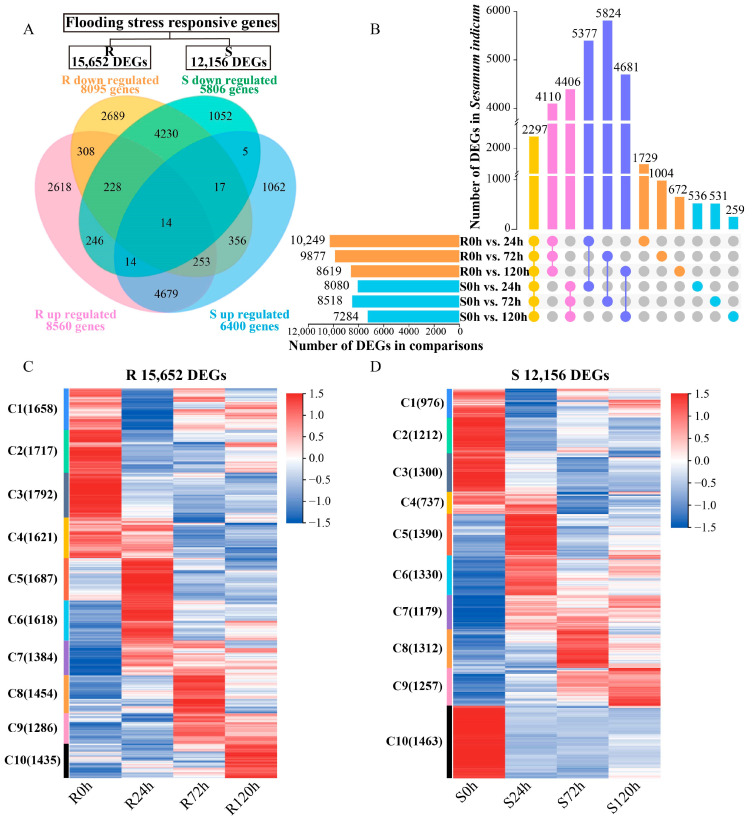
Differentially expressed genes (DEGs) in sesame under waterlogging stress at different times. (**A**). Venn diagram showing the intersection of up- and downregulated DEGs in R and S sesame. (**B**). UpSet plot showing the intersection of DEGs between different comparisons. Yellow means DEGs under waterlogging stress in all groups, pink means DEGs only in R or S, purple means DEGs between R and S under the same waterlogging treatment time, orange means DEGs only in R under waterlogging stress, and blue mesns DEGs only in S under waterlogging stress. (**C**,**D**) Heatmaps showing genes differentially expressed at each time point in R (**C**) or S (**D**) before (0 h) and after (24, 72, and 120 h) waterlogging treatment. A total of 15,652 DEGs in R and 12,156 DEGs in S were analyzed via using k-means clustering. The color key represents the standardized gene expression levels from high (red) to low (blue).

To understand the stress response of sesame to waterlogging, R and S sesame samples were subjected to waterlogging treatment at different times (0, 24, 72, and 120 h) and analyzed via transcriptomics (Table S1). DEGs at the different times were identified using the criterion of *p* value ≤ 0.05 and |log2FoldChange| ≥ 0.0 (Tables S2 and S3). The gene expression of a processing time under waterlogging treatment (24, 72, and 120 h) meets this standard, and the gene is considered to be a DEG. As shown in Figure 2A, a total of 15,652 DEGs were identified in R, of which 8560 were upregulated and 8095 were downregulated. A total of 12,156 DEGs were identified in S, of which 6400 were upregulated and 5806 were downregulated. Moreover, 502 DEGs were upregulated in R but downregulated in S, and 640 DEGs were downregulated in R but upregulated in S. In addition, 5377, 5824, and 4681 DEGs were identified separately between R and S under waterlogging treatment at 24, 72, and 120 h, respectively, and 2297 DEGs were significantly different between R and S at all treatment times, as shown in Figure 2B. The expression analysis of the DEGs in R and S revealed that all the DEGs were divided into 10 clusters, and their expression data are shown in Figure 2C,D. The expression patterns of R and S were similar under waterlogging stress, but there were also differences. For example, the expression of the C10 cluster in R was significantly upregulated, whereas that in S was significantly downregulated. The different expression levels of these DEGs in R and S under waterlogging stress indicate that these DEGs may play important roles under waterlogging stress.

### 2.3. Analysis of DEGs Function Under Waterlogging Stress

**Figure 3:**
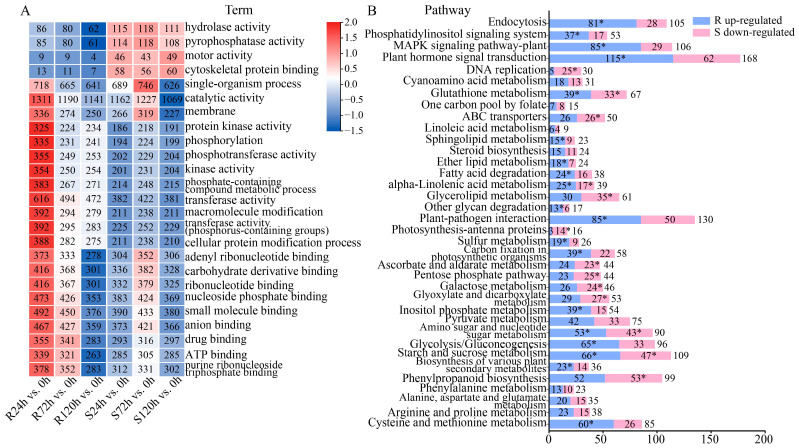
DEG functional analysis in sesame at different durations of waterlogging stress. (**A**). Gene Ontology (GO) analysis. Top 25 GO terms significantly enriched in DEGs upregulated in R and downregulated in S. (**B**). Kyoto Encyclopedia of Genes and Genomes (KEGG) analysis. KEGG pathways significantly enriched in DEGs upregulated in R and downregulated in S. * indicates significantly enriched pathways in R or S. The number indicates the number of DEGs.

To further explore the functions of the DEGs under stress and analyze the mechanism of sesame response to waterlogging stress, we performed Gene Ontology (GO) and Kyoto Encyclopedia of Genes and Genomes (KEGG) analyses on the upregulated DEGs in R and the downregulated DEGs in S (Figures S2–S4). The top 25 terms significantly enriched in the GO analysis are shown in Figure 3A. A comparison of the top 25 terms for the DEGs enriched in R and S at different waterlogging durations revealed that S was more enriched than R in hydrolase activity, pyrophosphatase activity, motor activity, and cytoskeletal protein binding and that R was significantly enriched in transferase activity, phosphotransferase activity, and protein kinase activity. R had more DEGs at 24 h, and S had more at 72 h. The results of the GO analysis revealed that catalytic activity and single-organism processes may play important roles in sesame’s response to waterlogging stress. The KEGG analysis of the DEGs revealed that 36 pathways were significantly enriched, as shown in Figure 3B. The plant hormone signal transduction pathway was enriched with the most DEGs. In addition, the glutathione metabolism, amino sugar and nucleotide sugar metabolism, starch and sucrose metabolism, and alpha-linolenic acid metabolism pathways were significantly enriched in both the DEGs upregulated in R and those downregulated in S. In summary, these pathways contribute to sesame response to waterlogging stress.

### 2.4. Weighted Gene Co-Expression Network Analysis (WGCNA) of Sesame Under Waterlogging Stress

**Figure 4:**
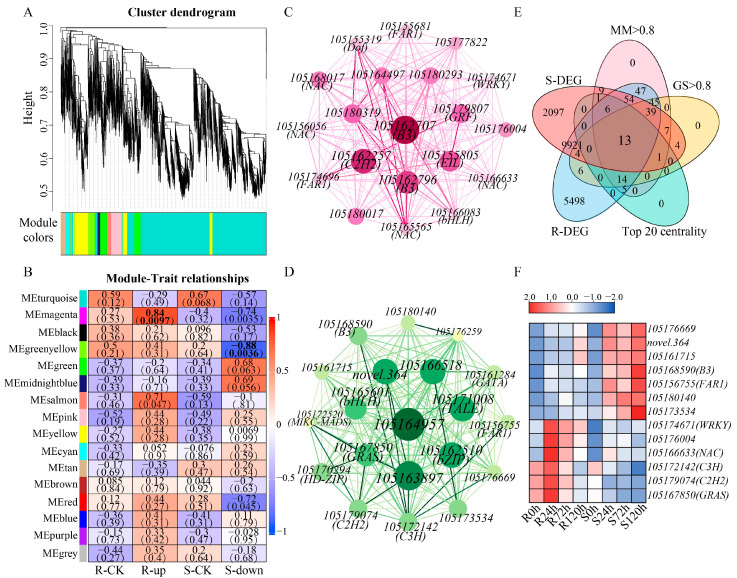
Weighted gene co-expression network analysis (WGCNA) of sesame under waterlogging stress. (**A**). Cluster dendrogram. Cluster dendrogram of the top 8000 genes with the greatest variation among the genes upregulated in R and those downregulated in S. Each vertical line in the dendrogram represents a gene. All genes are clustered into 16 modules, which are represented by turquoise, magenta, black, green-yellow, green, midnight blue, salmon, pink, yellow, cyan, tan, brown, red, blue, purple, and grey, respectively. (**B**). Correlation heatmap between 16 modules and the genes upregulated in R and those downregulated in S. The upper numbers in the table are correlations, and the lower numbers in parentheses are significant. Bold is the strongest correlation. Positive and negative correlations are indicated in red and blue, respectively. (**C**,**D**). The correlation network of the magenta (**C**) and green-yellow (**D**) modules. Magenta represents magenta module, and green-yellow represents green-yellow module. The gene network was constructed via WGCNA, and each node represents a gene; the connecting line (edge) between genes represents the co-expression correlation. The size of the gene font and the depth of the color are determined by the co-expression correlation. The higher the co-expression correlation, the larger the gene font and the deeper the color. On the contrary, the smaller the gene font, the lighter the color. The top 20 genes combined with the centrality and number of edges were visualized via Cytoscape. (**E**). Venn diagram of the hub genes. (**F**). Heatmaps showing hub gene expression before and after waterlogging stress.

To further identify the genes related to waterlogging stress, WGCNA was performed on the DEGs upregulated in R and those downregulated in S. According to the gene expression data, a total of 16 co-expression modules were obtained, which were turquoise, magenta, black, green-yellow, green, midnight blue, salmon, pink, yellow, cyan, tan, brown, red, blue, purple, and grey; the genes of the gray module (totaling six) did not belong to any module (Figure 4A). The number of genes per module ranged from 64 (midnight blue) to 3098 (turquoise), indicating that there were differences between modules. In addition, we generated a correlation heatmap to analyze the relationships between the modules and the genes upregulated in R and downregulated in S under waterlogging stress (Figure 4B). The magenta module and the green-yellow module were significantly positively correlated with the upregulated genes in R and negatively correlated with the downregulated genes in S, respectively. The genes in the magenta (Figure 4C) and green-yellow (Figure 4D) modules were screened by centrality, and the genes in the top 20 centrality were visualized, including *NAC*, *WRKY*, *bHLH*, *FAR1*, *B3*, *C2H2*, and *bZIP*, were visualized. In the magenta and green-yellow modules, 133 and 241 genes were identified as candidate hub genes based on |gene significance (GS)| > 0.8 and |module membership (MM)| > 0.8, respectively. According to the intersection of these candidate genes with the top 20 centrality genes and DEGs, 13 hub genes were identified, including *WRKY*, *NAC*, and *C2H2* (Figure 4E,F; Table S4). The hub genes may be key genes involved in regulating the response of sesame to waterlogging stress.

### 2.5. Differentially Expressed miRNAs (DEMis) Analysis of Sesame Under Waterlogging Stress

**Figure 5:**
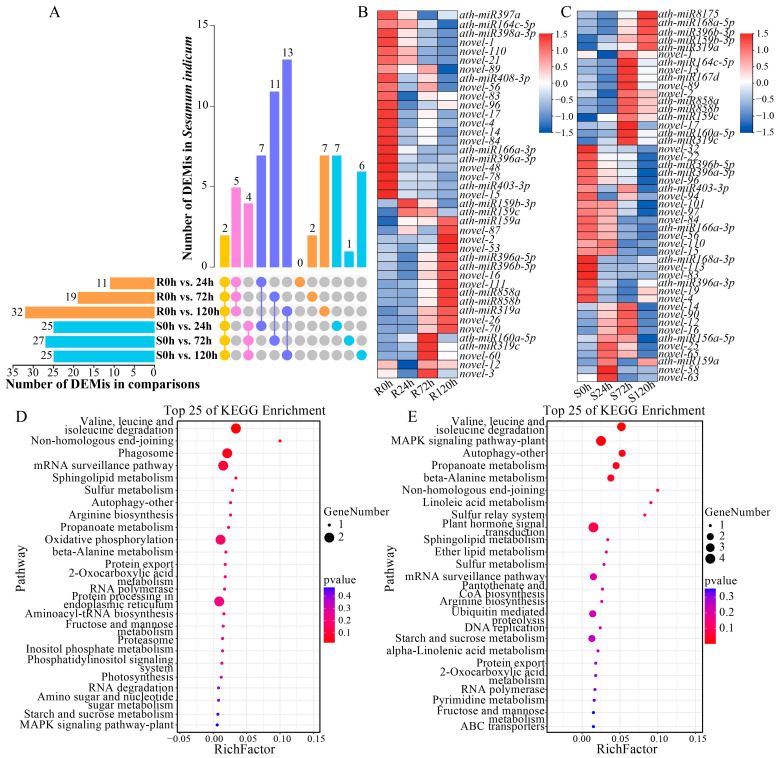
Differentially expressed miRNAs (DEMis) in sesame at different waterlogging treatment times. (**A**). UpSet plot showing the intersection of DEMis between different comparisons. Yellow means DEMis under waterlogging stress in all groups, pink means DEMis only in R or S, purple means DEMis between R and S under the same waterlogging treatment time, orange means DEMis only in R under waterlogging stress, and blue mesns DEMis only in S under waterlogging stress. (**B**,**C**). Heatmaps showing the DEMis that were differentially expressed in R (**B**) and S (**C**). (**D**,**E**). KEGG analysis. Top 25 KEGG pathways enriched by DEMi target genes in R (**D**) and S (**E**).

To further analyze the response of sesame to waterlogging stress, R and S plants were subjected to waterlogging treatment at different times (0, 24, 72, and 120 h), and the expression of miRNAs was analyzed (Table S5). The DEMis at different waterlogging treatment times were determined with *p* ≤ 0.05 and |log2FoldChange| ≥ 0.0 (Tables S6 and S7). With increasing waterlogging treatment time, the number of DEMis in R increased gradually, from 11 at 24 h to 19 at 72 h and finally to 32 at 120 h, for a total of 41 DEMis at all treatment times (Figure 5A). Among the 41 DEMis in R, 19 were upregulated and 23 were downregulated, and the expression of novel-3 changed significantly at different treatment times (Figure 5B). In S, the number of DEMis changed from 25 to 27 and finally returned to 25 with increasing waterlogging treatment time from 24 h to 72 h to 120 h, and there were 47 DEMis at all treatment times (Figure 5A). Among the 47 DEMis in S, 26 were upregulated and 23 were downregulated, and the expression of novel-2 and novel-89 differed at different treatment times (Figure 5C). The study also revealed that *ath-miR159c* and *ath-miR319a* were significantly different in both R and S at the three treatment times and were significantly upregulated, indicating that they may be related to sesame response to waterlogging stress.

A KEGG analysis of the target genes of DEMis was performed to further predict the function of DEMis (Figure 5C,D). In R, the oxidative phosphorylation, valine, leucine and isoleucine degradation pathways, etc., were enriched (Figure 5D). The MAPK signaling pathway and plant hormone signal transduction pathways were enriched in S (Figure 5E). In summary, these pathways may be involved in the regulatory effect of sesame on waterlogging stress.

### 2.6. Construction of DEMi and DEG Co-Expression Networks

**Figure 6:**
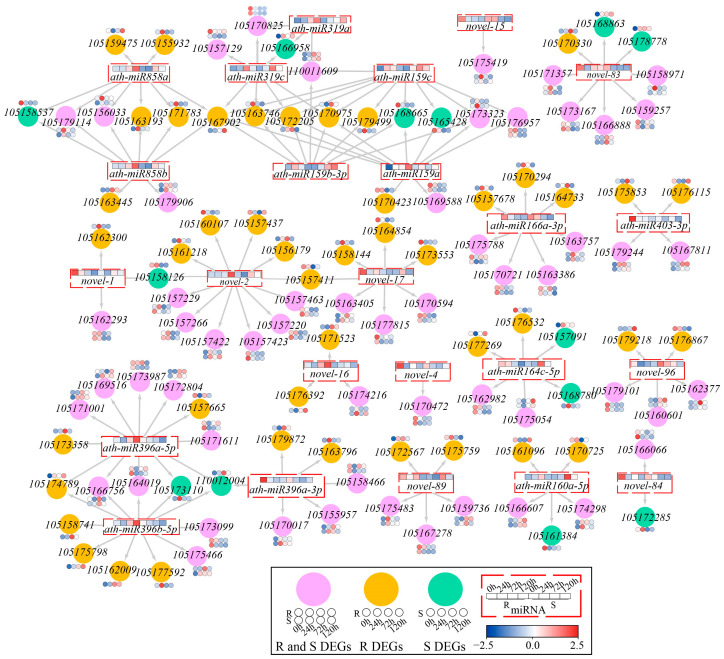
DEMi and DEG correlation network. The yellow circles in the network indicate the DEGs in R, the green circles indicate the DEGs in S, and the pink circles indicate the common DEGs both in R and S. The red dotted boxes in the network indicate the DEMis both in R and S. The arrow points to DEGs that miRNA can regulate.

To further explore the relationships between miRNAs and genes in sesame, the target genes of DEMis under waterlogging stress were predicted, and the target genes were screened with DEGs (Table S8). The integration analysis of 41 DEMis in R and 47 DEMis in S revealed that a total of 28 DEMis were enriched in both R and S. The target genes of 28 DEMis were predicted, and 192 target genes were identified, of which 114 were DEGs. Based on the DEMis and DEGs, 153 target gene pairs were ultimately identified, including 24 DEMis and 114 DEGs, of which 56 DEGs were significantly different in both R and S, and a co-expression network relationship was constructed (Figure 6). An analysis of the expression levels revealed that in R, the expression of most DEMis changed significantly at 72 h and 120 h, and their target genes also presented similar expression trends. In S, the DEMis changed significantly at 24 h, and the expression of the target genes also changed in the early stage of stress. These findings indicate that miRNAs can regulate the expression of target genes in response to waterlogging stress. A network diagram revealed that miRNAs had a negative regulatory effect on their target genes and could regulate multiple target genes. Moreover, target genes can also be regulated by multiple miRNAs, thus forming a miRNA–target gene regulatory network and participating in the response to stress.

### 2.7. Analysis of Sesame Metabolites Under Waterlogging Stress

**Figure 7:**
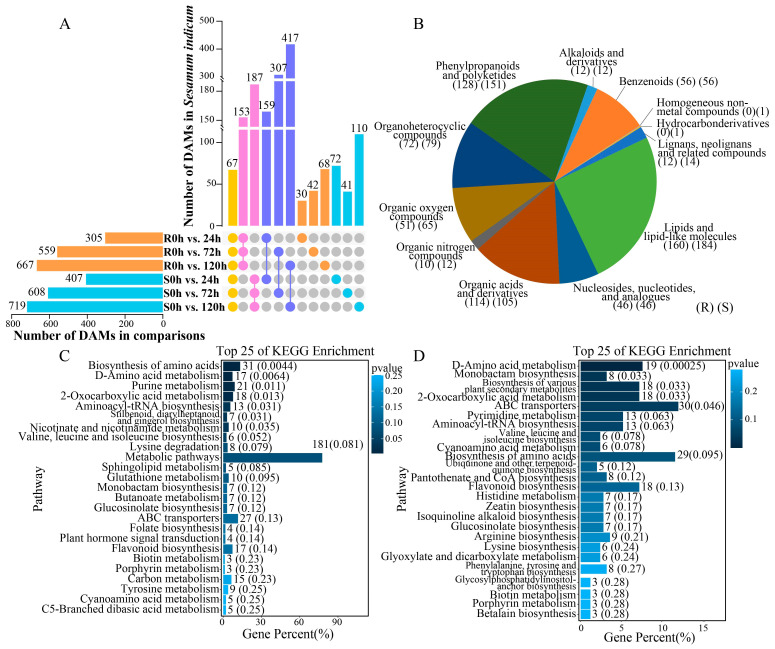
Analysis of differentially accumulated metabolites (DAMs) in sesame under waterlogging stress. (**A**). UpSet plot showing the DAMs under waterlogging stress. Yellow means DAMs under waterlogging stress in all groups, pink means DAMs only in R or S, purple means DAMs between R and S under the same waterlogging treatment time, orange means DAMs only in R under waterlogging stress, and blue mesns DAMs only in S under waterlogging stress. (**B**). Classification of DAMs. (**C**,**D**). KEGG analysis. Top 25 KEGG pathways enriched by DAMs in R (**C**) and S (**D**).

To explore the regulatory mechanism of sesame in response to waterlogging stress, the metabolites of sesame were analyzed and identified via metabolomics technology. A total of 896 differentially accumulated metabolites (DAMs) were identified in R, including 305 at 24 h, 559 at 72 h, and 667 at 120 h under waterlogging stress (Figure 7A, Table S9). A total of 1036 DAMs were identified in S, including 407 at 24 h, 608 at 72 h, and 719 at 120 h under waterlogging stress (Figure 7A, Table S10). DAMs were classified into 12 categories, including lipids and lipid-like molecules, phenylpropanoids and polyketides, organic acids and derivatives, organoheterocyclic compounds, benzenoids, organic oxygen compounds, nucleosides, nucleotides, and analogues; lignans, neolignans and related compounds; alkaloids and derivatives; organic nitrogen compounds and homogeneous non-metal compounds; and hydrocarbon derivatives, of which homogeneous non-metal compounds and hydrocarbon derivatives were found only in S (Figure 7B). According to the number of DAMs in each classification, only the numbers of organic acids and derivative metabolites in R were greater than in S, indicating that these compounds may be related to the response to waterlogging stress. In addition, 67 DAMs were significantly different in both R and S at all stress times, indicating that they may play an important role in the sesame response to stress.

To understand the functions of the metabolites, a KEGG analysis was performed (Figure 7C,D, Figures S5 and S6). In R, the D-amino acid metabolism, purine metabolism, aminoacyl-tRNA biosynthesis, and nicotinate and nicotinamide metabolism pathways were significantly enriched (Figure 7C). More than 10 DAMs were also enriched in ABC transporters, flavonoid biosynthesis, and glutathione metabolism (Figure 7C). In S, D-amino acid metabolism, the biosynthesis of various plant secondary metabolites, and ABC transporters were significantly enriched, and more than 10 DAMs were enriched in pyrimidine metabolism, aminoacyl-tRNA biosynthesis, and flavonoid biosynthesis (Figure 7D). The results of the KEGG analysis of the DAMs revealed that pathways related to metabolite metabolism and transport may be involved in the regulation of sesame under waterlogging stress.

### 2.8. KEGG Pathway Analysis of the Integrated DEGs and DAMs

To further analyze the relationships between genes and metabolites under waterlogging stress, we integrated the DEGs and DAMs of R and S for KEGG analysis. The plant hormone signal transduction, glutathione metabolism, and glyoxylate and dicarboxylate metabolism pathways were enriched, indicating that the above pathways may be involved in the response of sesame to waterlogging stress (Tables S11 and S12).

**Figure 8:**
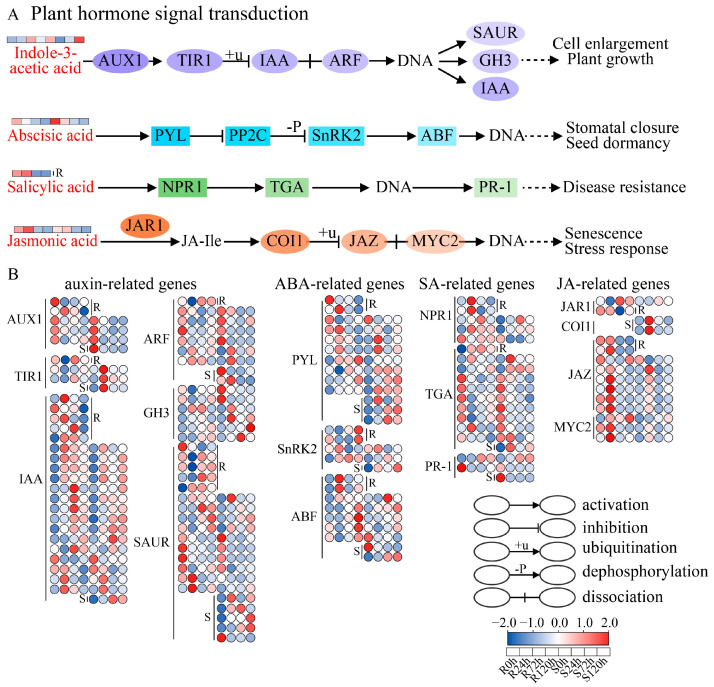
Plant hormone signal transduction in sesame under waterlogging stress. (**A**). DAMs related to the plant hormone signal transduction pathway. Red represents the DAM. (**B**). DEGs related to the plant hormone signal transduction pathway. Heatmaps showing the expression of DEGs or DAMs at different waterlogging treatment times.

In the plant hormone signal transduction pathway, four DAMs were enriched, including indole-3-acetic acid (IAA), ABA, SA, and JA, which are involved in the auxin, ABA, SA, and JA signal transduction pathways, respectively (Figure 8A). In the auxin signal transduction pathway, the accumulation of indole-3-acetic acid in R tended to increase first and then decrease, whereas in S, it tended to decrease first and then increase (Figure 8A). The expression of *AUX1* was significantly downregulated after stress (Figure 8B). The expression of *TIR1* in R first decreased but then increased after stress, whereas in S it was significantly upregulated first and then downregulated. *ARF* tended to be significantly downregulated in both R and S, *IAA* and *GH3* tended to be upregulated after stress treatment, the expression of the *SAUR* gene tended to be significantly downregulation, and the change in gene expression was more obvious in S. These findings indicate that sesame adapts to waterlogging stress by reducing auxin signal transduction. In the ABA signal transduction pathway, ABA tended to decrease, and the expression of genes such as *PYL*, *SnRK2*, and *ABF* significantly increased after stress, indicating that sesame responds to the regulation of waterlogging stress by increasing the transduction of ABA signals (Figure 8A,B). In the SA signal transduction pathway, SA only significantly changed in R and was significantly downregulated (Figure 8A). The expression of *NPR1* was upregulated after stress treatment, and the expression of *TGA* and *PR-1* was downregulated after treatment, which reduced the transduction of SA signals, thus affecting the response to waterlogging stress (Figure 8B). In the JA signaling pathway, JA was significantly downregulated (Figure 8A). *JAR1* and *COI1* were significantly upregulated after stress treatment (Figure 8B). The expression of *JAZ* and *MYC2* was upregulated first and then downregulated after stress, and the expression in R was significantly greater than that in S. These findings indicate that sesame may respond to stress by regulating JA signal transduction in the early stage of waterlogging stress.

**Figure 9:**
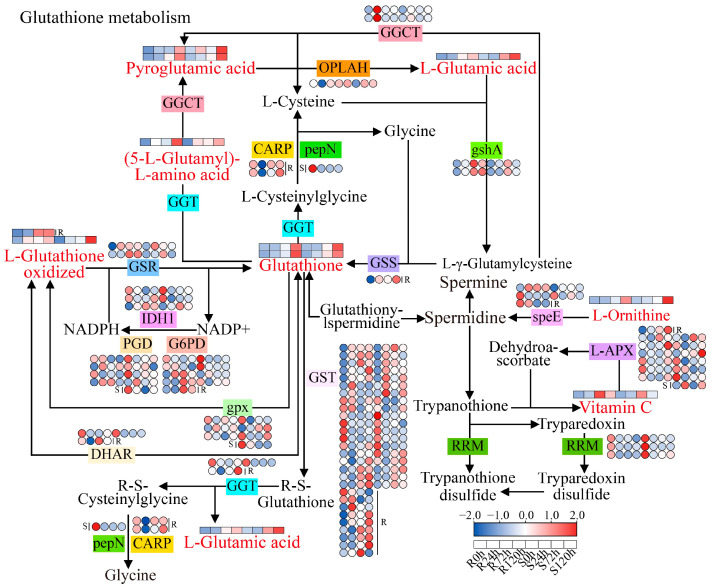
Glutathione metabolism pathway in sesame under waterlogging stress. Red represents the DAM. Heatmaps showing the expression of DEGs or DAMs at different waterlogging durations.

In the glutathione metabolism pathway, 10 DAMs and 74 DEGs were enriched (Figure 9). At the metabolic level, the enriched metabolites included L-glutathione, oxidized glutathione, L-ornithine, vitamin C, L-glutamic acid, L-glutathione oxidized, pyroglutamic acid, (5-L-glutamyl)-L-amino acid, L-pyroglutamic acid, and glutathione. The accumulation of all the metabolites was significantly increased under waterlogging stress. At the gene level, the expression of *GGCT*, *gshA*, *GSR*, *GSS*, *GST*, *speE*, and *L-APX* was significantly upregulated under stress, whereas the expression of *pepN*, *IDH1*, *PGD*, *G6PD*, *DHAR*, *GGT*, *RRM*, and *gpx* was significantly downregulated under stress; the expression of *CARP* and *OPLAH* was significantly downregulated in the early stage of stress (24 h) and upregulated in the later stage of stress (72 and 120 h). Comprehensive DAM and DEG analyses revealed that with increasing waterlogging duration, the expression of *GSR*, *GSS*, *GST*, and other genes also gradually increased. Moreover, glutathione, L-glutamic acid, L-glutathione oxidized, and other metabolites gradually accumulated, especially glutathione, which accumulated most significantly at 120 h.

**Figure 10:**
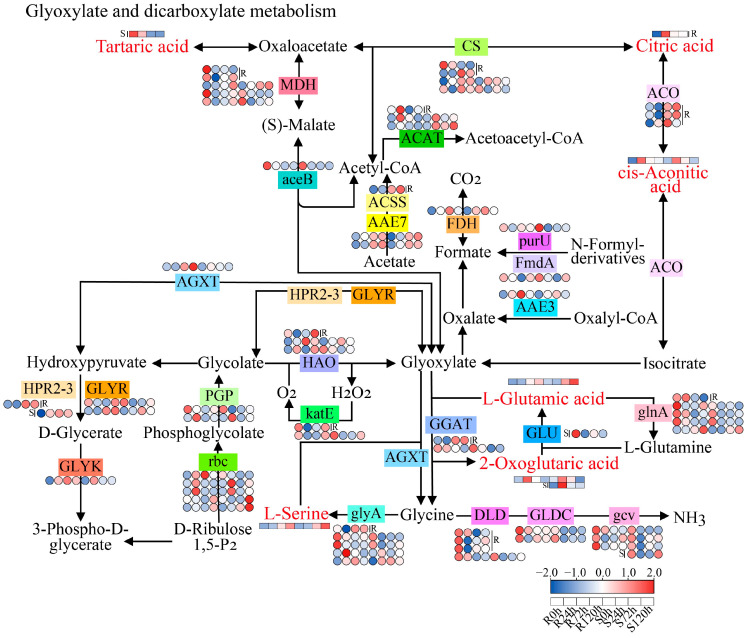
The glyoxylate and dicarboxylate metabolism pathway in sesame under waterlogging stress. Red represents the DAM. Heatmaps showing the expression of DEGs or DAMs at different waterlogging treatment times.

In the glyoxylate and dicarboxylate metabolism pathway, 7 DAMs and 64 DEGs were enriched (Figure 10). At the metabolic level, the content of tartaric acid changed significantly only in S, and its accumulation was significantly downregulated. The accumulation of L-glutamic acid and L-serine was significantly increased in the late stage of stress (72 and 120 h), and the accumulation of citric acid, cis-aconitic acid, and 2-oxoglutaric acid was increased in the early stage of stress. The accumulation of citric acid significantly changed only in R. At the gene expression level, the expression of *MDH*, *aceB*, *purU*, *PGP*, *DLD*, *GLDC*, *gcv*, *glnA*, *GLU*, and *GGAT* tended to decrease under waterlogging stress. The expression of *CS*, *ACO*, *ACAT*, *ACSS*, *HPR2-3*, *AAE7*, *FDH*, *AGXT*, *AAE3*, *HAO*, *GLYK*, *rbc*, and *glyA* was upregulated under waterlogging stress, and most of the genes were upregulated in the later stage of stress, among which *ACO* changed significantly only in R. The expression of *FmdA* and *KatE* was significantly downregulated at 24 h and upregulated at 120 h. A comprehensive study of DAMs and DEGs revealed that with increasing waterlogging duration, the expression of genes such as *CS* and *ACO* gradually increased in R, whereas the accumulation of the metabolite cis-aconitic acid first increased but then decreased, and the accumulation reached a maximum at 24 h.

### 2.9. qRT-PCR Validation

**Figure 11:**
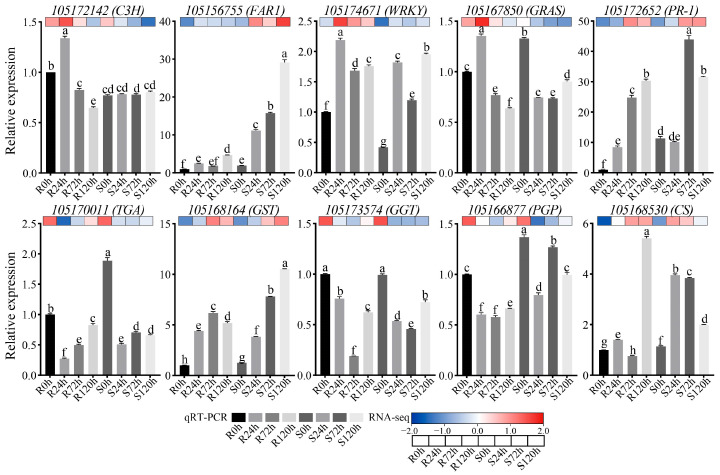
qRT-PCR analysis of analysis of DEGs. The mRNAs were isolated from the roots of R and S with waterlogging treatment, respectively. The *α-tubulin* gene was chosen as the internal control. Student’s *t* test was used for significance analysis, and different lowercase letters indicate *p* < 0.05. Bar means the average ± SD, n = 3.

To test the accuracy of the experimental data in this study, we performed a qRT-RCR analysis. Ten genes were randomly selected from the DEGs for qRT-RCR analysis. The expression pattern determined via qRT-PCR analysis under waterlogging stress was consistent with the results of the transcriptomic analysis, as shown in Figure 11 and Table S13. The results show that the transcriptome sequencing results are accurate.

## 3. Discussion

Water is essential for plant growth; however, excessive water can cause waterlogging stress. Studies have shown that waterlogging stress causes an anoxic environment around the roots of plants, hinders the transport and absorption of nutrients and the accumulation of toxic substances, inhibits the growth and development of plants, and even causes premature senescence and wilting [11]. Therefore, understanding the regulatory mechanism of waterlogging stress is highly important. Sesame is an important oil crop with drought tolerance, but it is relatively sensitive to water stress in the critical period of growth and development [23]. Research on sesame drought tolerance is relatively extensive, and relatively few studies have focused on waterlogging stress. In this study, the response of sesame to waterlogging stress was studied. Combined with the results of multi-omics sequencing, the key genes, miRNAs, metabolites, and important regulatory pathways of sesame in response to waterlogging stress were revealed.

### 3.1. Transcriptome Analysis

A total of 15,652 and 12,156 DEGs were identified in the two sesame genotypes R and S, respectively. The results of the GO analysis revealed that the DEGs were most enriched in catalytic activity, and the results of this study were consistent with those of rye (*Secale cereale*) under waterlogging stress [27]. Through the KEGG analysis, it was found that plant hormone signal transduction, glutathione metabolism, and starch and sucrose metabolism were significantly enriched with DEGs. Previous studies have shown that plant hormones and energy conversion play important roles under waterlogging stress, and this result was further verified in our study [28]. For the screening of key candidate genes, this study used the WGCNA method, which has been reported in many studies [29,30]. A total of 13 hub genes were identified, which is highly important for the study of the sesame response to waterlogging stress.

### 3.2. Small RNA Sequencing Analysis

The regulatory effect of miRNAs on the plant response to stress is accomplished by regulating the expression of their target genes [31]. Previous studies on miRNAs under waterlogging stress revealed that miRNAs such as *miR528*, *miR408*, *miR319*, *miR167*, *miR164*, *and miR159* can respond to stress [15]. In this study, 41 and 47 DEMis were identified in R and S, respectively, and 28 DEMis were significantly different between the two genotypes. In the study, it was found that in the early stage of waterlogging stress (24 h), the miRNA in S changed significantly, while the change of miRNA in R increased with the increase in stress time. It shows that the tolerance of sesame to waterlogging stress is affected by time. Even the waterlogging-tolerant genotype will be harmed to a certain extent and may even be more harmful after waterlogging exceeds its tolerance range [26]. Through the combined analysis of DEMis and DEGs, 153 pairs of key target gene pairs were identified, including 24 DEMis and 114 DEGs. The expression of miRNAs such as *miR858*, *miR319*, *miR159*, and *miR396* significantly changed after waterlogging treatment, and these results are consistent with studies on the response of maize to short-term waterlogging treatment [32]. In addition to the miRNAs involved in waterlogging stress, 10 new miRNAs were identified, suggesting that these miRNAs may also play important roles in waterlogging stress.

### 3.3. Metabolomics Analysis

Metabolomics analysis involves the analysis of endogenous metabolites in organisms, including their types, quantities, and changes in response to external factors [33]. Compared with other omics analysis methods, metabolomics analysis can better reflect the overall information of organisms under external stimulation. Studies have shown that, under waterlogging treatment, plants generally inhibit growth by slowing metabolic reactions, such as carbohydrate explanation and energy supply, to respond to stress [34]. This conclusion was fully verified in the present study. A total of 896 and 1036 DAMs were identified in R and S, respectively, by metabolomics sequencing, among which lipids and lipid-like molecules, phenylpropanoids and polyketides, and organic acids and derivatives were the three most enriched categories. Flavonoid biosynthesis and vitamin B6 metabolism pathways play important roles in rapeseed (*Brassica napus*) waterlogging stress [35]. In this study, in addition to the flavonoid biosynthesis pathway, ABC transporters and glutathione metabolism were also significantly enriched, suggesting that these genes may play important roles in waterlogging stress.

### 3.4. Plant Hormone Signal Transduction Pathway in Response to Sesame Waterlogging Stress

Plant hormones play an important role in the whole life cycle of plants in the face of biotic and abiotic stresses. In this study, many DEGs related to the plant hormone signaling pathway were also identified after waterlogging stress, and four DAMs were identified, namely, ABA, IAA, JA, and SA. Studies have shown that when the content of ABA increases, its receptor PYR1/PYL/RCAR can bind to it and act on PP2C, thereby inhibiting the protein phosphatase activity of PP2C and the dephosphorylation of SnRK2 [36]. In contrast, SnRK2 activates downstream transcription factors to trigger the ABA response through its own phosphorylation [36]. In this study, the ABA content significantly decreased, and the expression of the *PYL*, *SnRK2*, and *ABF* genes increased after waterlogging stress, which was consistent with the above results, indicating that sesame plays a role in waterlogging stress via the ABA response. Auxin is regulated by establishing a concentration gradient during plant growth and development; that is, low concentrations promote and high concentrations inhibit auxin [37]. A study of the response of *Solanum dulcamara* to waterlogging stress revealed that the production of adventitious roots requires auxin transport [38]. In our study, the changes in the auxin contents of the two genotypes were quite different. In R, the auxin content first increased but then decreased, whereas in S, it first decreased and then increased, but both tended to increase overall, indicating that sesame adapted to waterlogging stress possibly by regulating the auxin content. With respect to SA and JA, studies on the response of soybean to floods have shown that SA and JA may participate in the stress response by acting as detoxification hormones [11]. In our study, the changes in SA and JA contents were consistent, both of which first increased but then decreased, but the expression of related genes in the SA signal transduction pathway showed the opposite trend [39]. In summary, plant hormones play important roles in the sesame response to waterlogging stress, and a variety of hormones play a role in combination.

### 3.5. Glutathione Metabolic Pathway in Response to Sesame Waterlogging Stress

When plants are under waterlogging stress, a large amount of ROS are produced in the body, which causes membrane lipid peroxidation, destroys the cell structure, and affects normal growth [40]. In this situation, plants initiate the antioxidant system in the body to remove excessive ROS to maintain the metabolic balance in plants [8]. Glutathione, a nonenzymatic antioxidant, is involved in the detoxification of H_2_O_2_ to maintain ROS homeostasis in plant cells [41]. Studies related to waterlogging tolerance in soybeans have shown that glutathione is highly important for identifying excessive ROS and maintaining cell homeostasis [42]. In this study, the content of glutathione was significantly increased under waterlogging stress. Through the combined analysis of transcriptomics and metabolomics, it was also found that the accumulation of metabolites such as L-glutamic acid and L-glutathione oxidized increased significantly after waterlogging stress. In addition, many DEGs were enriched in the glutathione metabolic pathway, and the expression of most genes was upregulated. The results of this study are consistent with the results of citrus waterlogging tolerance [8]. These findings indicate that the removal of excessive ROS by increasing the glutathione content maintains cell homeostasis and plays a role in sesame response to waterlogging stress.

### 3.6. Glyoxylate and Dicarboxylate Metabolism Pathway in Response to Sesame Waterlogging Stress

Under waterlogging stress, some plants also accelerate energy metabolism to adapt to stress. A study of peach (*Prunus persica*) revealed that energy metabolism and anaerobic respiration under anaerobic or anoxic conditions play important roles in waterlogging stress [41]. In our study, through multi-omics conjoint analysis, the glyoxylate and dicarboxylate metabolism pathways were significantly enriched. The glyoxylate cycle involves the transformation of lipids to sugars in the process of plant metabolism, which plays a very important role in the growth and development of plants [43]. Organic acids such as citric acid and cis-aconitic acid were enriched in the glyoxylate and dicarboxylate metabolism pathways, and their accumulation increased after waterlogging treatment. *FDH* was also enriched and upregulated after waterlogging treatment. Studies on legume nodules have shown that the accumulation of *FDH* is related to hypoxia [44]. In summary, the results showed that sesame may promote the conversion of lipids to sugars and anaerobic respiration through glyoxylate and dicarboxylate metabolism pathways in response to waterlogging stress.

## 4. Materials and Methods

### 4.1. Plant Materials and Waterlogging Treatment

In the early stage, the waterlogging tolerant variety HHZ1 (R) and the sensitive variety Zhengzhi 13 (S) were identified by screening sesame. In this study, R and S were used as experimental materials, and pot experiments were carried out. Firstly, the two types of sesame seeds were cultured in the incubator for 24 h. After the seeds were exposed to white, the seeds with the same bud length were selected and evenly planted in the pot. After emergence, the cotyledons grew to 1 pair of true leaves stage for thinning, 2–3 pairs of true leaves stage for seedling, and there were 5 seedlings per pot. Pot seedlings with similar growth vigor were selected when 4 pairs of true leaves were fully expanded at seedling stage. The pots were directly placed into a turnover box with a depth of 16 cm, and the water surface was irrigated to exceed 1 cm of the soil surface for waterlogging treatment. The intact roots of R and S sesame seedlings were collected at 0, 24, 72, and 120 h after waterlogging treatment, and 3 replicate samples were taken for each treatment time. After quick freezing in liquid nitrogen, it was temporarily stored in a refrigerator at −80 °C for subsequent analysis.

### 4.2. Physiological Phenotype of Sesame Under Waterlogging Treatment

The physiological indicators of frozen samples stored at −80 °C were evaluated and included POD and APX activity and MDA, GSH, SS, SP, and Pro contents. The Pro and MDA content was determined by Anee’s methods [45]. The contents of APX and GSH were measured by integrating Paradiso’s and Huang’s methods [46,47].

### 4.3. Transcriptome Sequencing Analysis

Total RNA was extracted for subsequent analysis via the TRIzol method, and the integrity of the RNA was measured via an Agilent 2100 bioanalyzer (Agilent Technologies, Santa Clara, CA, USA). Oligo dT magnetic beads were used to enrich total RNA with mRNAs. After fragmentation, the cDNA was synthesized, its ends were modified, and it was purified to construct a cDNA library. Sequencing was performed via the Illumina platform of Novogene. Raw reads were processed by fastp software (v0.23.4) to obtain clean reads [48]. HISAT2 (v2.0.5) was used to quickly and accurately align the clean reads with the reference genome, and new genes were predicted by using StringTie (v1.3.3b) [49,50]. In accordance with the results of alignment with the reference genome, the read numbers of the genes were obtained via featureCounts (v1.5.0-p3) software, and the expression level of the gene FPKM was subsequently obtained [51,52]. DEGs were identified using DESeq2 (1.20.0) software with *p* value ≤ 0.05 and |log2FoldChange| ≥ 0.0 as the standard for differential gene screening. GO functional enrichment analysis and KEGG pathway enrichment analysis of the DEGs were performed via clusterProfiler (3.8.1) software, and genes with *p* value < 0.05 were considered significantly enriched [53,54]. WGCNA of the DEGs was performed via TBtools (v2.025), and the candidate genes among the hub genes were visualized via Cytoscape (3.10.1) [55]. The accuracy of transcriptomic analysis was analyzed using Pearson correlation coefficients and Principle component analysis (Figures S7 and S8) [56].

### 4.4. Small RNA (sRNA) Sequencing Analysis

Total RNA was used as the starting sample, and cDNA was synthesized via reverse transcription via the special structure of the 3′ and 5′ ends of small RNA. After RCR amplification, purification and fragment selection, a small RNA library was constructed. The 18–40-bp library was sequenced with an Illumina sequencer using the SE50 strategy from Novogene. Clean reads were obtained after quality control of the raw reads. The length of the clean reads was screened, and the length of the plant sRNAs ranged from 18–30 nt, of which micro-RNA (miRNAs) were concentrated from 21–22 nt [57]. The screened sRNAs were aligned to the reference genome by Bowtie (1.0.1), the known miRNA was annotated according to the miRBase 22.0 database by miRDeep2 (mirdeep2_0_0_5), and new miRNAs were analyzed by the miRNA prediction software miREvo (v1.1) [58]. The expression levels of known and new miRNAs were statistically analyzed and normalized to the TPM. DESeq2 (1.24.0) software was used to screen DEMis with *p* value ≤ 0.05 and |log2FoldChange| ≥ 0.0 as the standard, and TBtools (v2.025) was used to visualize their expression [55]. The target genes of the miRNAs were predicted via psRobot (psRobot_v1.2) software, and their functions were analyzed by KEGG analysis [59]. Cytoscape (3.10.1) software was used to visualize the DEMi-DEG regulatory network.

### 4.5. Metabolomics Sequencing Analysis

This study is based on liquid chromatography–mass spectrometry (LC–MS) technology for untargeted metabolomics research, completed by Novogene [60]. The raw data were used to perform a simple screening of metabolites via Compound Discoverer 3.3 (CD3.3, Thermo Fisher, Waltham, MA, USA), and then secondary identification of metabolites was performed by comparing the mzCloud (https://www.mzcloud.org/, accessed on 4 September 2024), mzVault, and MassList databases. Finally, metabolites with a coefficient of variance (CV) of less than 30% were used for subsequent analysis [61]. KEGG database (http://www.genome.jp/kegg/, accessed on 25 September 2024) annotation was performed on the identified metabolites to understand the functional characteristics. The screening of DAM was mainly based on variable importance in the projection (VIP), fold change (FC), and *p* value [62]. The thresholds were set as VIP > 1.0, *p* value < 0.05, and FC > 1.2, or FC < 0.833.

### 4.6. qRT-PCR Verification of Genes

Primer (v5.0) software was used to design the primers for qRT-RCR for randomly selected genes. The data were standardized to the expression of *α-tubulin*, and the differences were compared via the 2^−ΔΔCT^ method [63].

### 4.7. Statistical Analysis

One-way ANOVA processing was used as a phenotypic difference significance analysis method for sesame under waterlogging treatment (0, 24, 72, and 120 h). Student’s *t* test was used to test the significant difference in gene expression under waterlogging treatment (0, 24, 72, and 120 h). One-way ANOVA and Student’s *t* test were performed using IBM SPSS Statistics (version 21) software.

## 5. Conclusions

In this study, the transcriptome, small RNA sequencing, and metabolomics of sesame roots and the physiological phenotype of sesame under waterlogging stress were analyzed in detail. The study found that the content of various plant hormones in sesame changed significantly, indicating that it can respond to waterlogging stress by regulating plant hormone signal transduction pathway. Moreover, face to waterlogging stress, glutathione metabolic pathway of sesame also changed significantly, which promoted the synthesis of antioxidants and improved the antioxidant activity to alleviate the harm caused by waterlogging. In addition, the study found that glyoxylate and dicarboxylate metabolism pathway of sesame were affected to a certain extent, which promoted the conversion of lipids to sugars and anaerobic respiration, which greatly increased the tolerance of sesame to waterlogging stress. In this study, we identified 13 hub genes, which will contribute to the development and breeding of new water-tolerant sesame varieties in the future. In conclusion, the results of this study provide a feasible idea and theoretical basis for further systematic and comprehensive explanation of the regulation mechanism of sesame on waterlogging stress.

## Data Availability

The datasets generated during and/or analyzed during the current study are available from the corresponding author upon reasonable request.

## Supplementary Materials

The following supporting information can be downloaded at: https://www.mdpi.com/article/10.3390/ijms26010351/s1.
