# Supplementary material for: Physiological and Multi-Omics Integrative Analysis Provides New Insights into Tolerance to Waterlogging Stress in Sesame (Sesamum indicum L.)
Source: Int J Mol Sci. 2025 Jan 3;26(1):351. doi: 10.3390/ijms26010351 (PMC11720211; doi:10.3390/ijms26010351)
Supplement: Supplementary file 1 [file ijms-26-00351-s001.zip › Supplemental Figures.pdf]

Content

Figure S1. MDA content of sesame under waterlogging stress. 1
Figure S2. GO analysis of sesame DEG under waterlogging stress...2
Figure S3. KEGG analysis of sesame DEG under waterlogging stress in R. 3
Figure S4. KEGG analysis of sesame DEG under waterlogging stress in S. 4
Figure S5. KEGG analysis of sesame DAM under waterlogging stress in R. 5
Figure S6. KEGG analysis of sesame DAM under waterlogging stress in S. 6
Figure S7. Pearson correlation coefficients of sesame under waterlogging stress. 7
Figure S8. Principle component analysis of sesame under waterlogging stress. 8

Figure S1. MDA content of sesame under waterlogging stress.

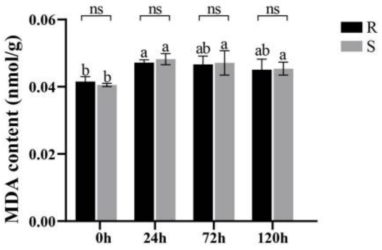

Figure S1. MDA content of sesame under waterlogging stress. R and S represent two types of sesame, R is waterlogging tolerance, S is waterlogging intolerance. Different lowercase letters indicate that there are significant differences in the same sesame waterlogging stress at different time ($p < 0.05$). ns indicates no significant change between the two sesame waterlogging stress at the same time.

Figure S2. GO analysis of sesame DEG under waterlogging stress.

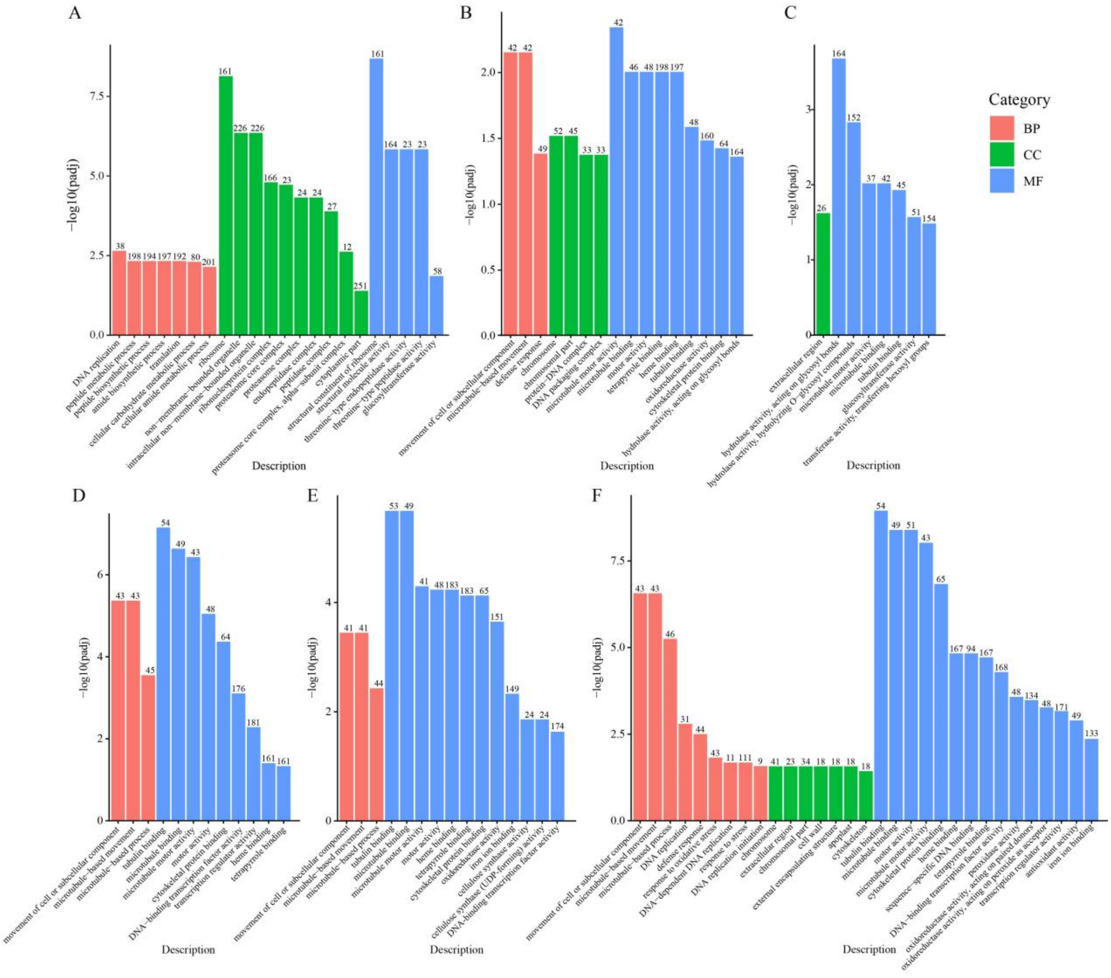

Figure S2. GO analysis of sesame DEG under waterlogging stress. A-C. Waterlogging stress treatment for 24 h (A), 72 h (B) and 120 h (C) of R. D-F. Waterlogging stress treatment for 24 h (D), 72 h (E) and 120 h (F) of S. The listed GO terms are all significantly enriched.

Figure S3. KEGG analysis of sesame DEG under waterlogging stress in R.

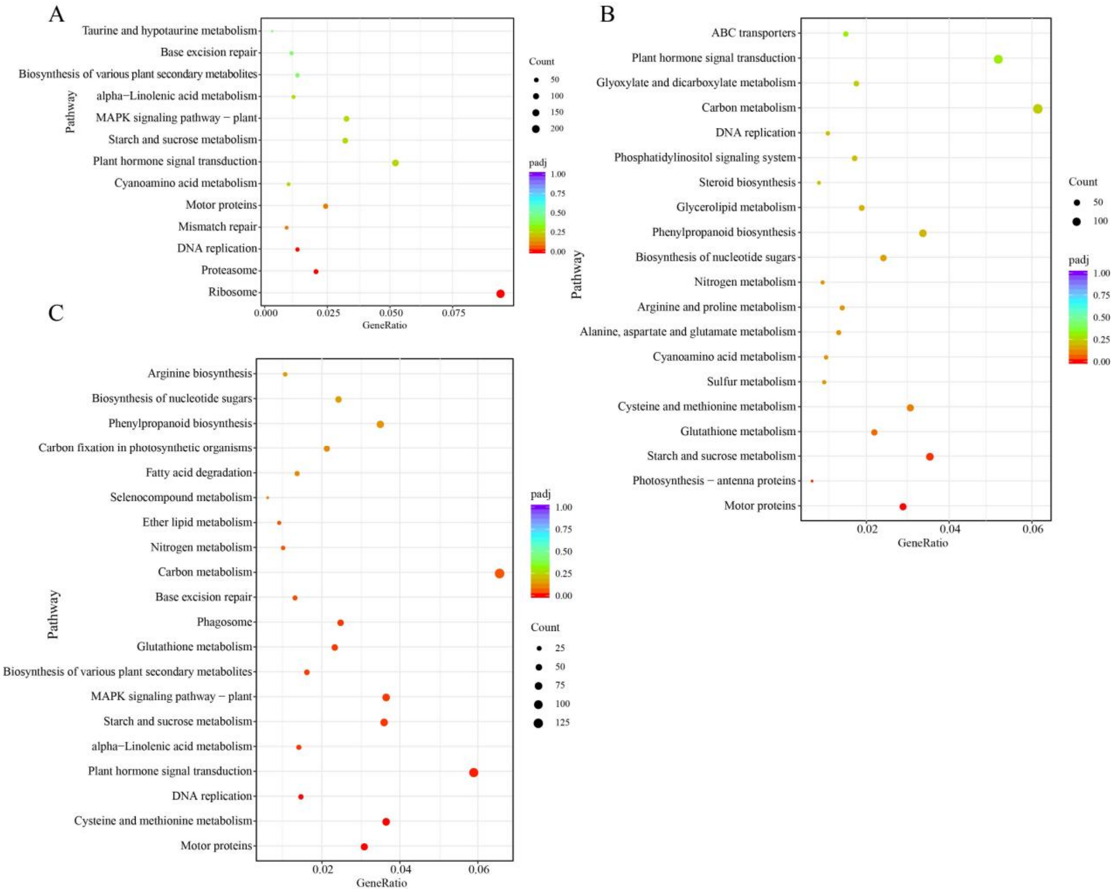

Figure S3. KEGG analysis of sesame DEG under waterlogging stress in R. A. Waterlogging stress treatment for 24 h. B. Waterlogging stress treatment for 72 h. C. Waterlogging stress treatment for 120 h. The pathways listed were significantly enriched, and more than 20 listed the top 20 pathways.

Figure S4. KEGG analysis of sesame DEG under waterlogging stress in S.

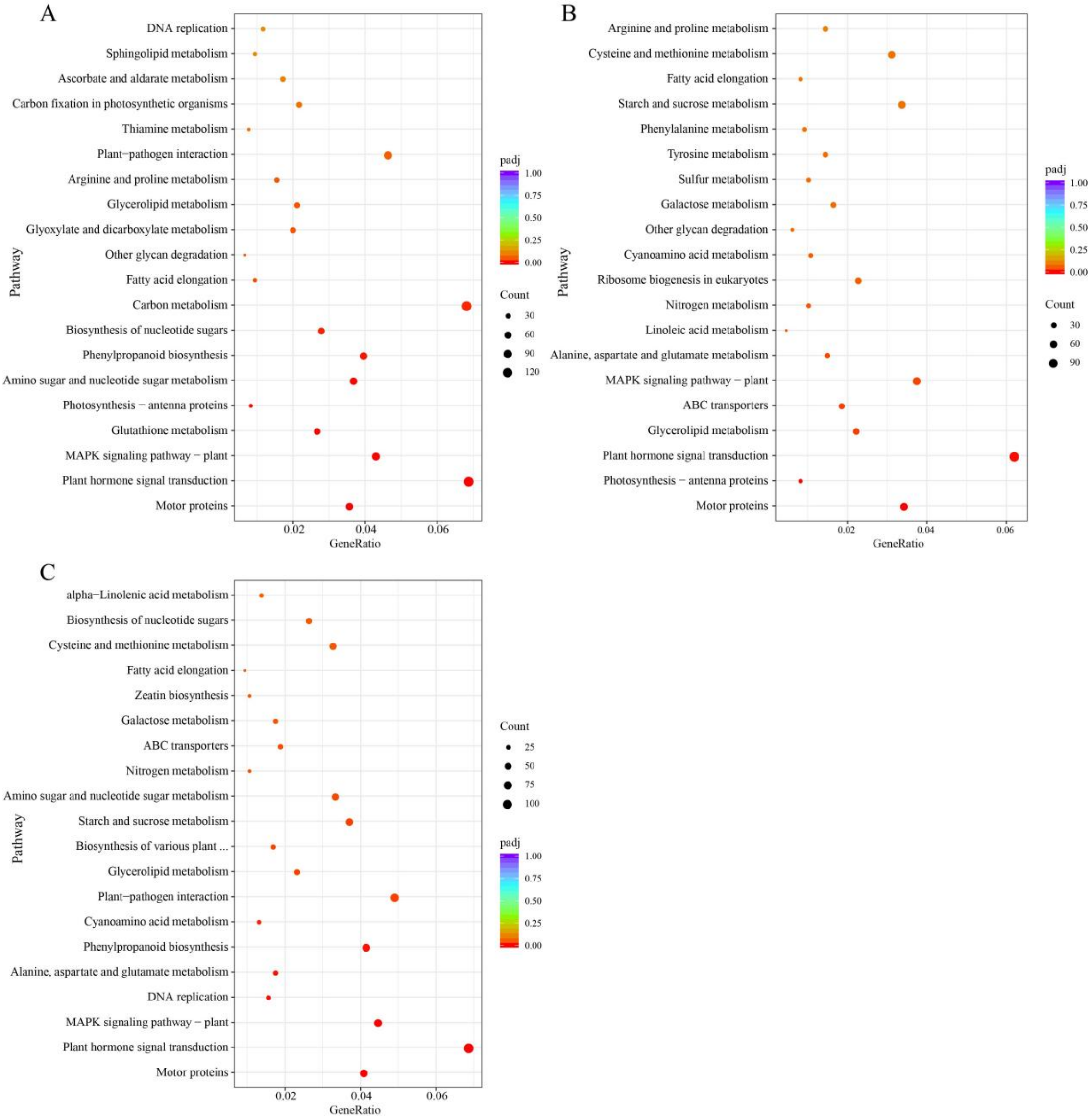

Figure S4. KEGG analysis of sesame DEG under waterlogging stress in S. A. Waterlogging stress treatment for 24 h. B. Waterlogging stress treatment for 72 h. C. Waterlogging stress treatment for 120 h. The pathways listed were significantly enriched, and more than 20 listed the top 20 pathways.

Figure S5. KEGG analysis of sesame DAM under waterlogging stress in R.

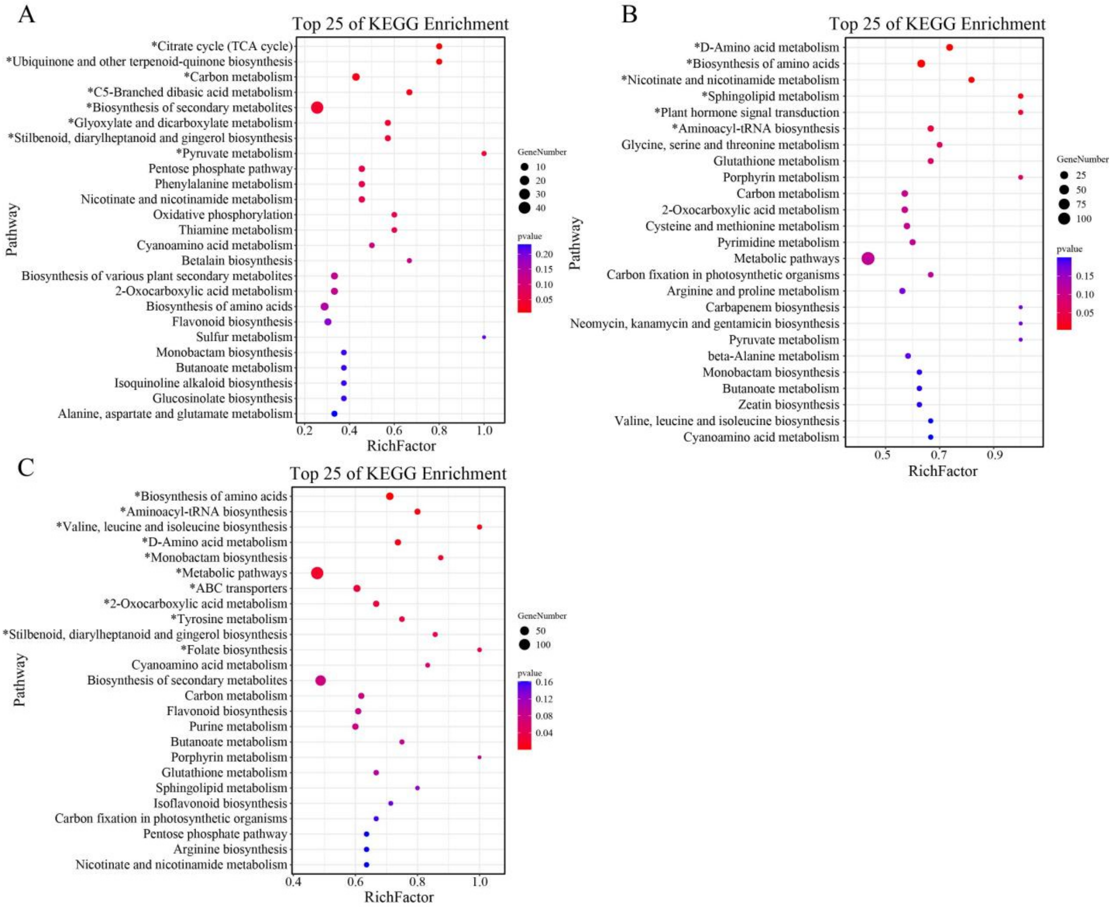

Figure S5. KEGG analysis of sesame DAM under waterlogging stress in R. A. Waterlogging stress treatment for 24 h. B. Waterlogging stress treatment for 72 h. C. Waterlogging stress treatment for 120 h. The pathways listed were the Top 25 enriched pathways, * means significant enrichment.

Figure S6. KEGG analysis of sesame DAM under waterlogging stress in S.

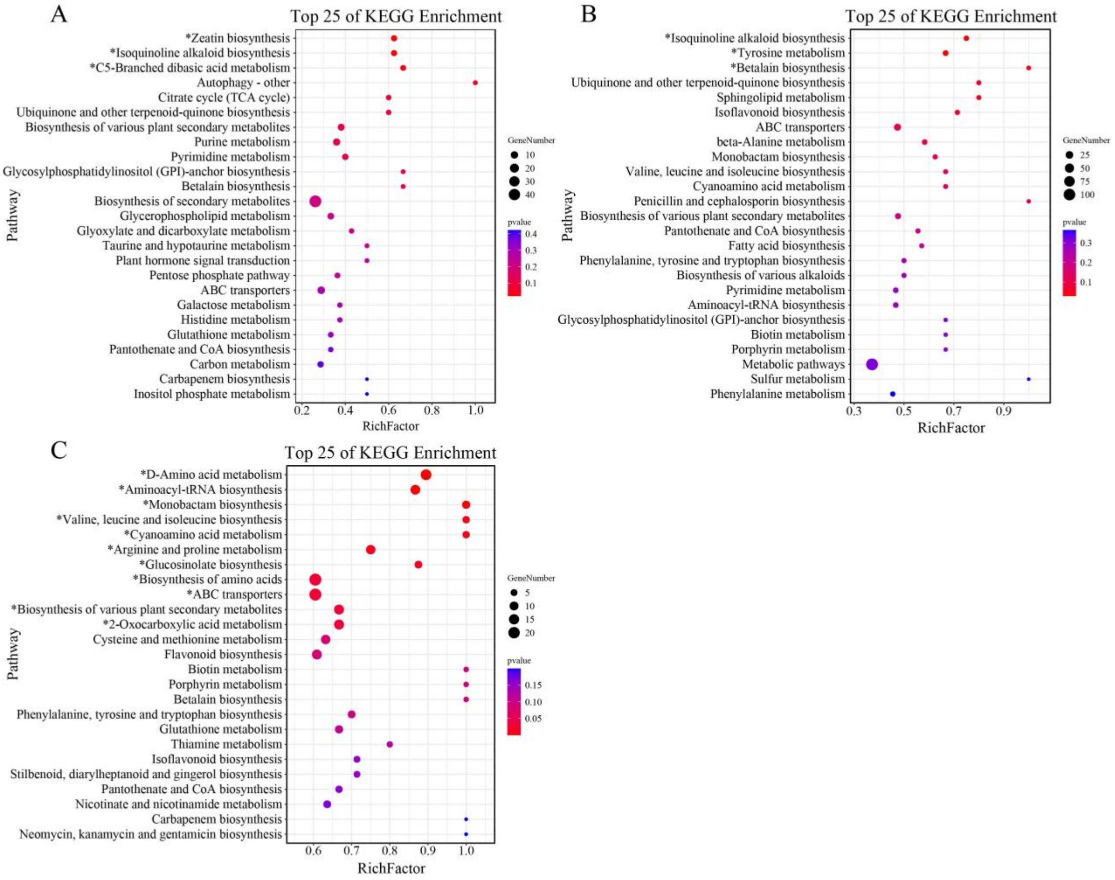

Figure S6. KEGG analysis of sesame DAM under waterlogging stress in S. A. Waterlogging stress treatment for 24 h. B. Waterlogging stress treatment for 72 h. C. Waterlogging stress treatment for 120 h. The pathways listed were the Top 25 enriched pathways, * means significant enrichment.

Figure S7. Pearson correlation coefficients of sesame under waterlogging stress.

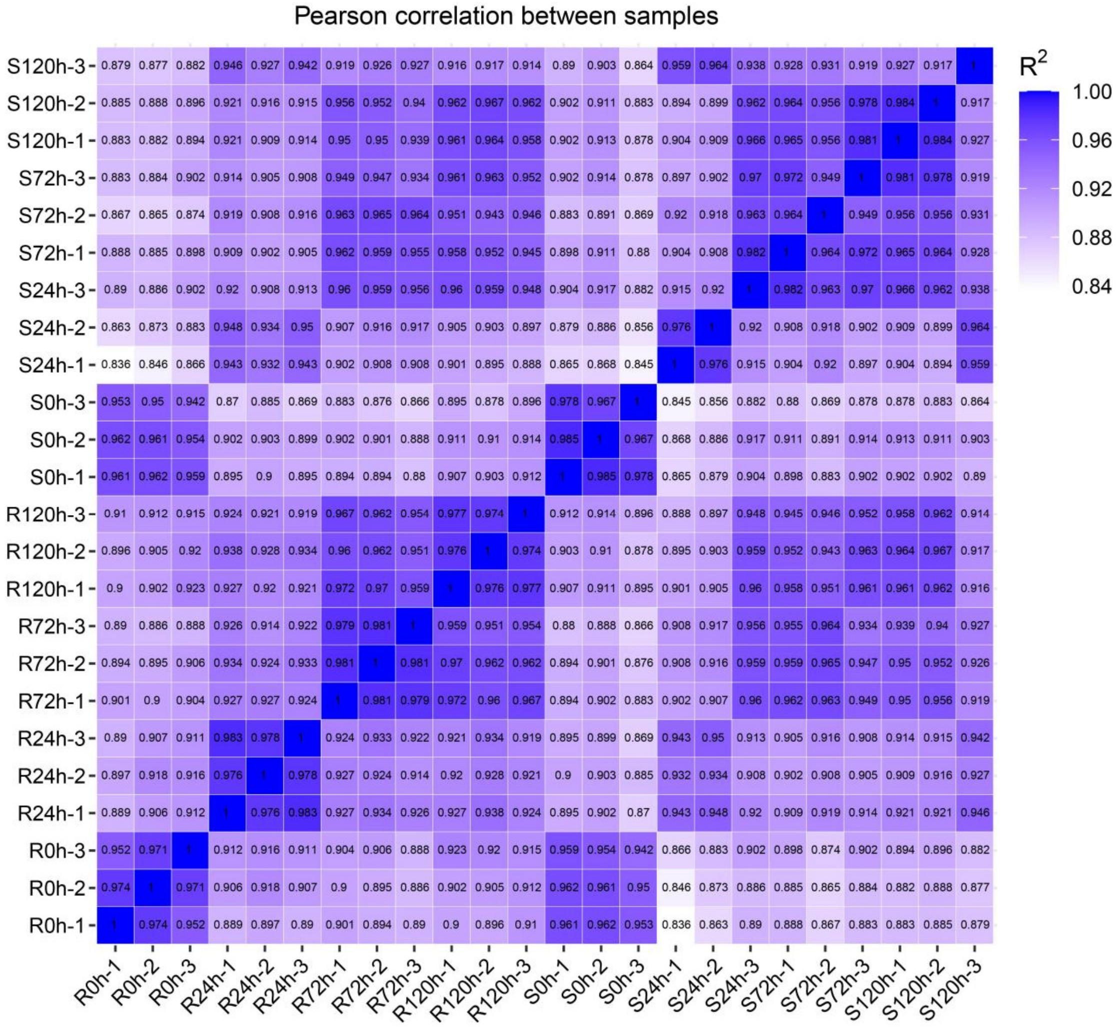

Figure S7. Pearson correlation coefficients of sesame under waterlogging stress. Three biological replicates for each time point.

Figure S8. Principle component analysis of sesame under waterlogging stress.

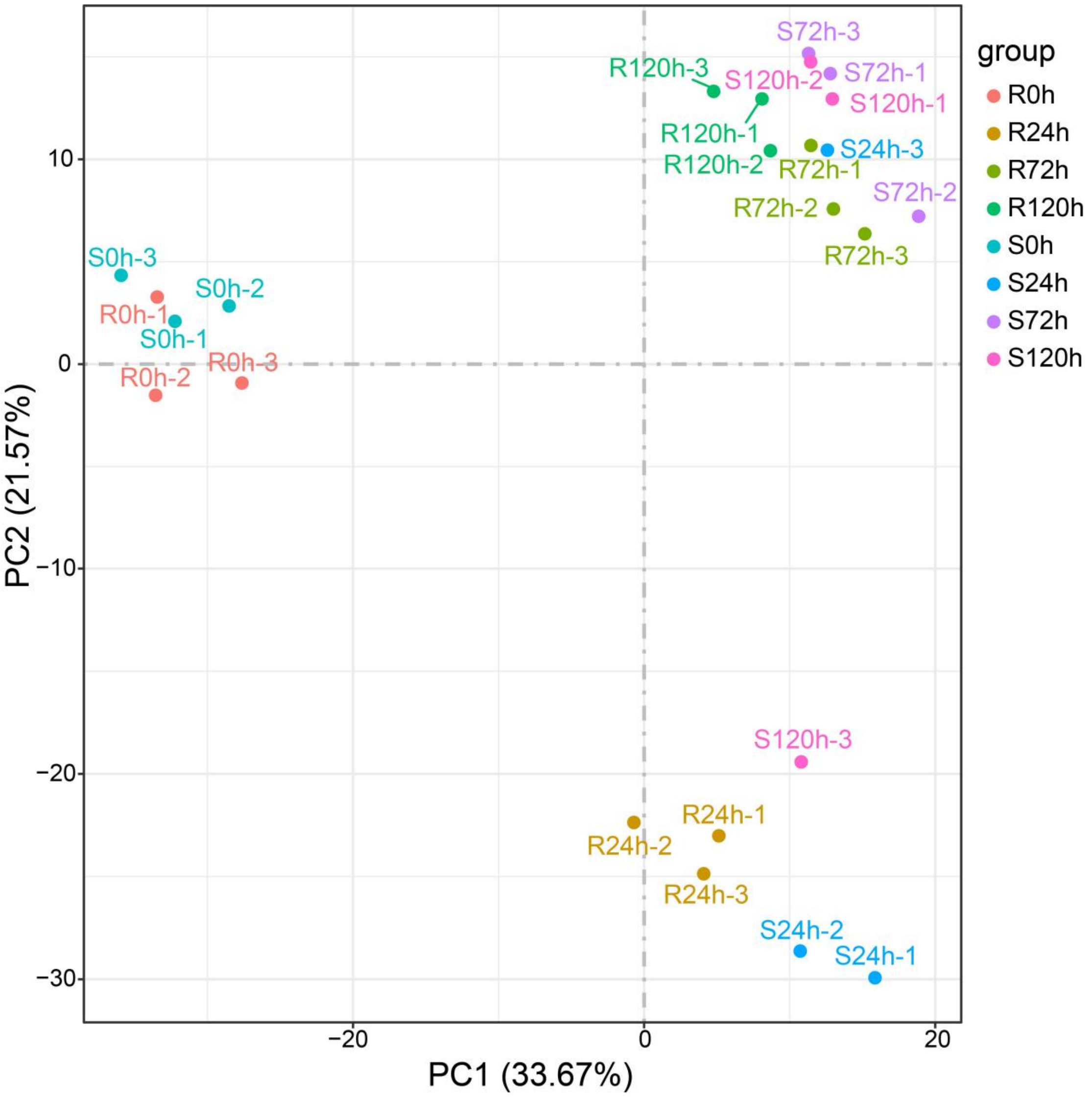

Figure S8. Principle component analysis of sesame under waterlogging stress. Three biological replicates for each time point.
